# Coronary steal phenomenon following right ventricle decompression and revascularization of atretic left coronary ostium: case report

**DOI:** 10.1186/s13019-021-01681-x

**Published:** 2021-10-13

**Authors:** Jacek Pająk, Maciej Aleksander Karolczak, Michał Buczyński, Wojciech Mądry, Darren James Grégoire, Piotr Weryński, Wanda Król-Jawień, Krzysztof Godlewski, Lidia Tomkiewicz-Pająk, Jacek Kuźma

**Affiliations:** 1 Department of Cardiac and General Pediatric Surgery, Medical University of Warsaw, Warsaw, Poland; 2 Department of Pediatric Cardiology, University Children’s Hospital of Krakow, Collegium Medicum, Jagiellonian University, Krakow, Poland; 3 Pediatric Cardiology and General Pediatrics Clinic, Medical University of Warsaw, Warsaw, Poland; 4 Department of Cardiac and Vascular Disease, Collegium Medicum, Jagiellonian University, John Paul II Hospital, Krakow, Poland

**Keywords:** Coronary artery atresia, Coronary fistula, Coronary revascularization, Fistula embolization

## Abstract

**Background:**

Coronary steal phenomenon and myocardial ischemia is a complication following decompression of a hypertensive right ventricle in patients with left coronary-cameral fistulae.

**Case presentation:**

We present a 12-year-old girl with a complex heart defect successfully operated on using a hybrid surgical-interventional approach to decompress the ventricle, embolize the fistula and reconstruct the atretic left coronary ostium.

**Conclusions:**

A novel hybrid strategy is the best solution for coronary-cameral fistulas reliant on high ventricular pressure at high risk for coronary steal phenomenon.

**Supplementary Information:**

The online version contains supplementary material available at 10.1186/s13019-021-01681-x.

## Background

Atresia of the left coronary artery ostium (ALCAO) is one of the rarest congenital coronary anomalies, in which a solitary right coronary artery supplies the entire myocardium. The myocardium may receive additional perfusion via fistulas from hypertensive ventricles [1, 2]. Unilateral coronary ostial atresia usually follows complex heart defects as in the presented case with pulmonary atresia causing critical stenosis with an intact ventricle septum and hypertensive right ventricle chamber. Patients with ALCAO and insufficient coronary perfusion may develop variable symptoms including chest pain, fatigue, dyspnea, arrhythmias, syncope, cardiac compromise and sudden cardiac death [3]. In most children with ALCAO the perioperative mortality is high, therefore heart transplant is the most viable option [4, 5]. We present a novel surgical-interventional hybrid alternative for successful restoration of anatomical and physiological coronary circulation complicated by myocardial ischemia due to coronary steal phenomenon.

## Case presentation

A 12-year-old girl with severe pulmonary valvar stenosis (PVS), atretic left main coronary artery ostium (ALCAO) and a wide fistula between the hypertensive right ventricle (RV) and the left coronary artery received surgical follow-up consultation.

The child had NYHA class II–III, episodic abdominal pain and exertional dyspnea. Physical examination showed good general condition, regular heart rate of 70 beats/min, loud systolic murmur of 4–5/6 on Levine scale with a thrill over the chest and jugular notch. Blood pressure was 100/60 mmHg, respiratory rate 14 breaths/min and SaO_2_ 96% on room air.

The heart defect was identified at birth: Transthoracic echocardiogram and heart catheterization revealed severe pulmonary stenosis, left coronary ostium atresia and a wide fistula connecting a hypertensive right ventricle with the left coronary artery.

Neonatal balloon pulmonary valvuloplasty decreased the severity of stenosis although the persistent suprasystemic RV pressure still provided sufficient coronary flow.

Corrective surgery was postponed due its complexity. Heart transplant remained a viable option due to high operative risk. The patient attended echocardiography for 12-year follow-up prior to admission to a high-level cardiac surgery center.

Normal sinus rhythm with right axis deviation and severe right ventricular hypertrophy were found on ECG. Roentgenogram showed cardiomegaly, pulmonary trunk dilatation and right atrium enlargement. TTE visualized 2^nd^ degree tricuspid valve regurgitation with a pressure gradient of 138 mmHg, and PVS pressure gradient of 148 mmHg. Hemodynamic studies recorded increased central venous (12 mmHg), LV end-diastolic (15 mmHg) and suprasystemic RV pressures (127/15 mmHg).

**Fig. 1:**
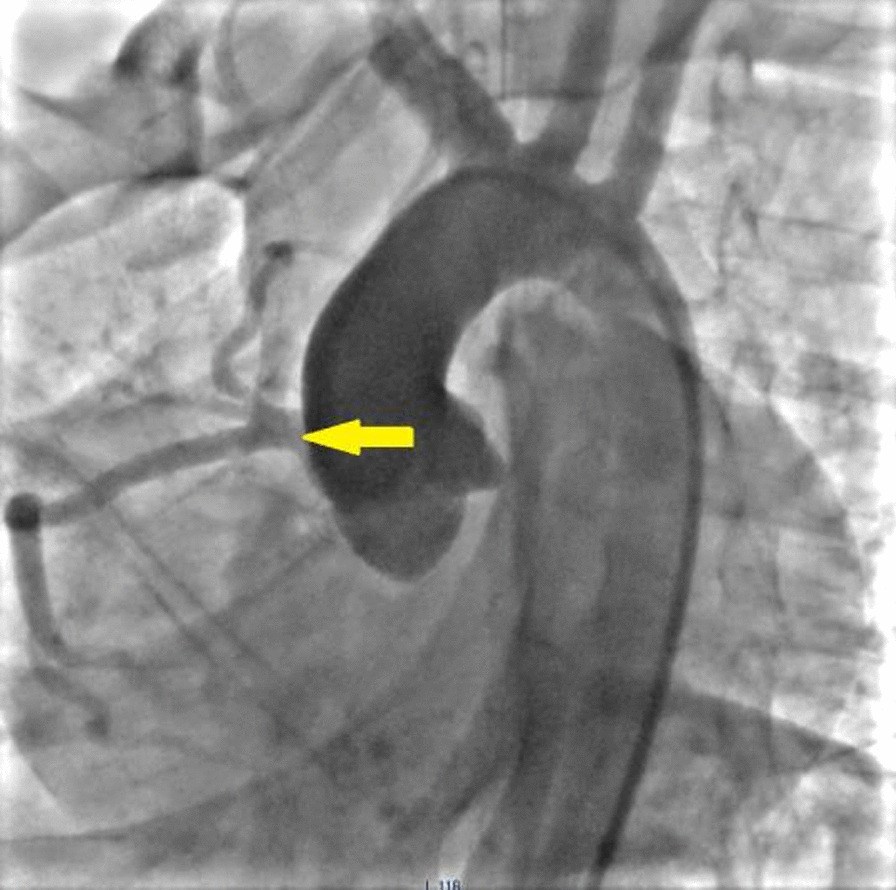
Aortography. Antero-posterior view showing wide right coronary artery (yellow arrow) with conal branch crossing right ventricular outlet tract. Absent left main coronary artery

**Fig. 2:**
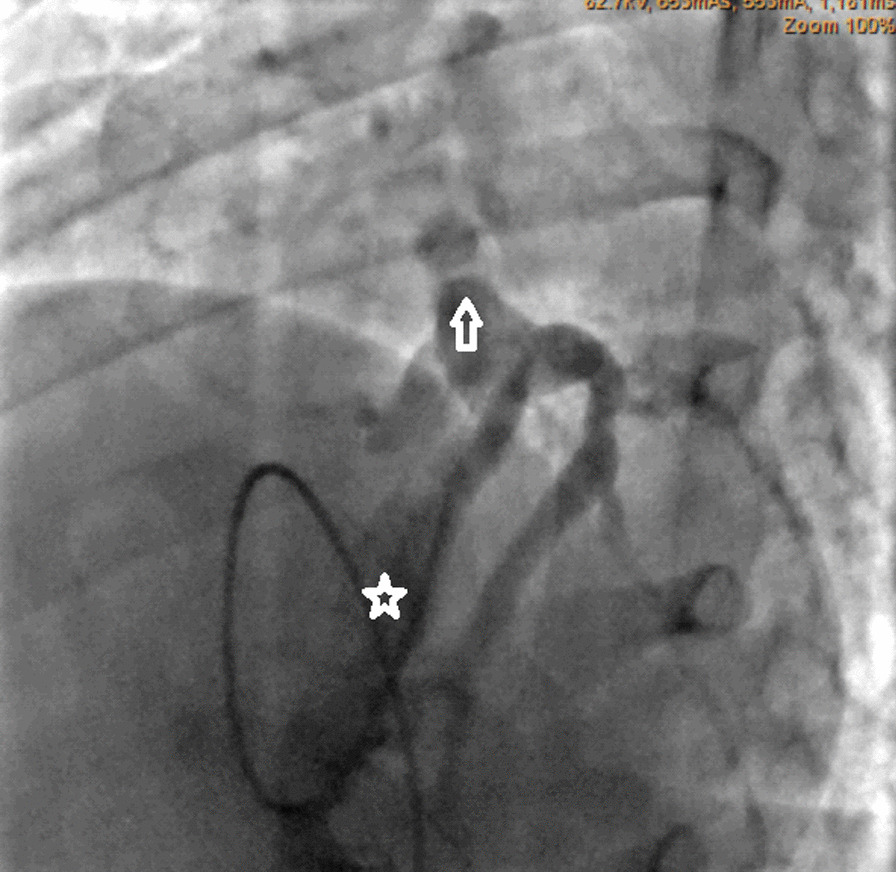
Selective fistula angiography (LAO 28°, Caudal 37°). Long and wide fistula (white star). Atresia of the left coronary artery ostium (white arrow)

**Fig. 3.**
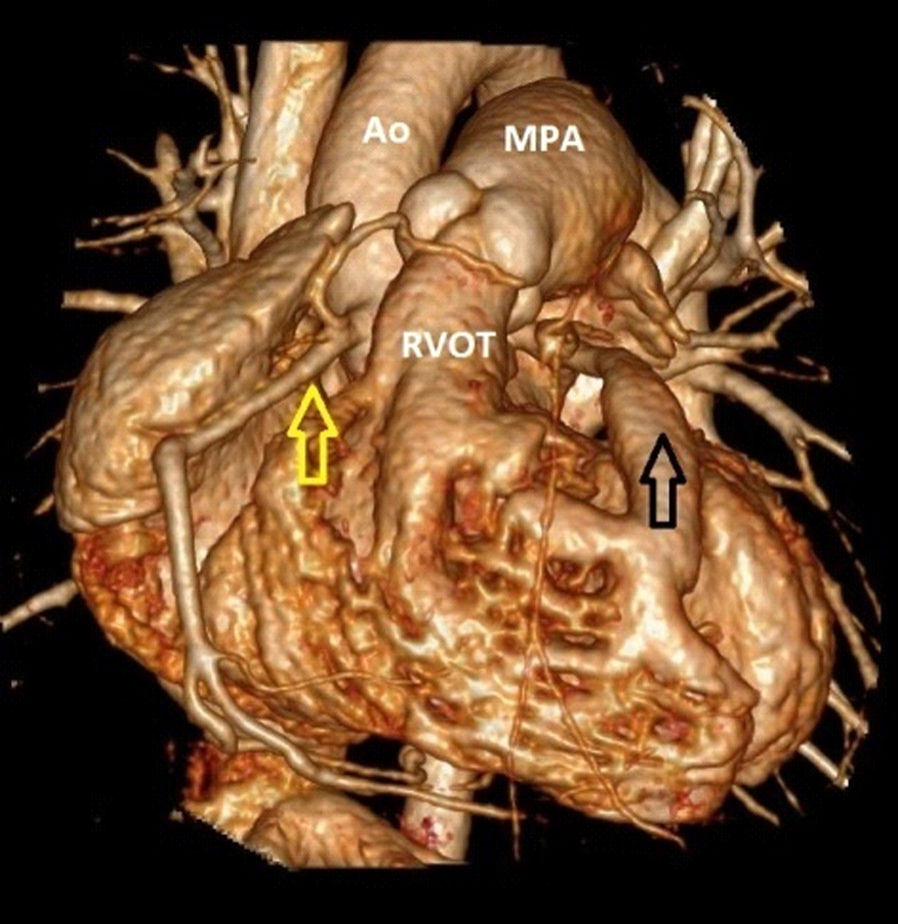
3D rendered computed tomography. A wide fistula (black arrow) with multiple branches emerging from a hypertrophic right ventricle. Wide right coronary artery (yellow arrow).* Ao* ascending aorta,* MPA* main pulmonary artery,* RVOT* right ventricular outlet tract

Angiographic studies illustrated wide RCA (5 mm) and atretic ostium of the LMCA (Fig. 1, Additional file 1: Video 1). Right ventriculography outlined severe pulmonary valvar stenosis with dome-shaped valve leaflets and post-stenotic pulmonary trunk dilatation. Furthermore, selective fistula angiography and 3D rendered Computed Tomography depicted ALCAO and a wide coronary fistula emerging from the hypertrophic RV (Figs. 2, 3; Additional file 2: Video 2, Additional file 3: Video 3). The fistula orifice sat superficially in the anterior interventricular groove. Multiple proximal and distal converging collateral anastomoses were present between the LMCA and RCA. The LMCA divided into the left anterior descending artery (LAD) and the circumflex artery (CX).

**Fig. 4:**
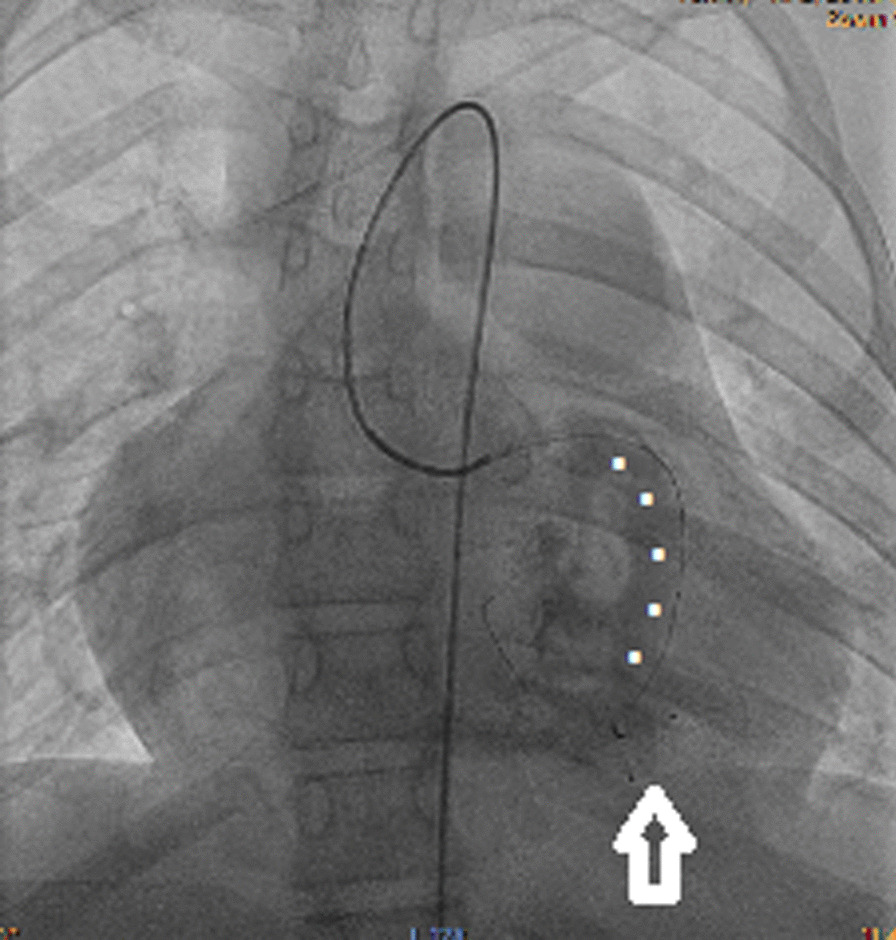
Selective left coronarography with patent fistula (white dots) and the steal phenomenon from the left coronary artery into the decompressed right ventricle. The occluder devices implanted during the operation (white arrow)

**Fig. 5:**
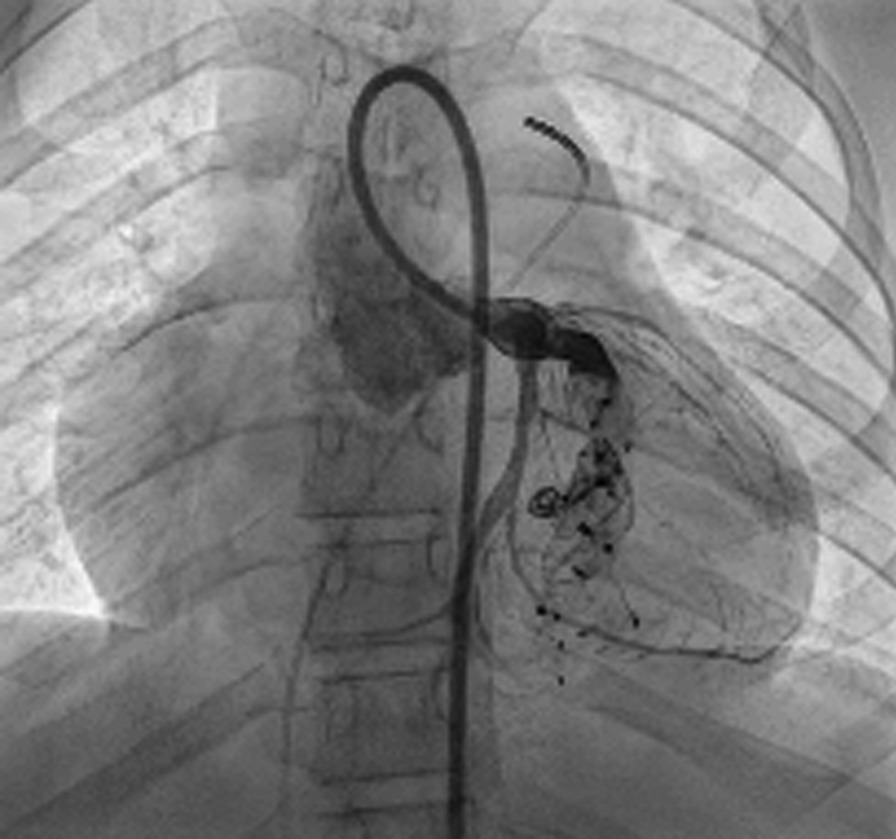
The fistula with multiple occluder devices and restored antegrade coronary flow

The patient was qualified for LMCA ostium reconstruction and simultaneous interventional fistula embolization. A sternotomy was performed with cross-clamped aorta and cannulated superior and inferior cava veins under 24 °C hypothermia on cardiopulmonary bypass which supported the patient. An infusion of cardioplegic solution to the RCA sustained electromechanical quiescence to asystole. Epicardial fat meant the fistula was not visible. The ascending aorta, bulb and proximal LMCA segment were precisely dissected. The ostium was atretic due to a fibrous diaphragm which was excised, and the coronary artery ostium reconstructed with a Biointegral patch. Next, a RVOT infundibulectomy was undertaken with pulmonary commissurotomy and partial resection of the dysplastic leaflets. The now reconstructed and patent LMCA ostium provided a conduit for a multipurpose 5-F catheter over a 0.025-inch hydrophilic steerable guidewire. The fistula branches were implanted with two 6 mm diameter Amplatzer Vascular Plugs II (St. Jude Medical, St Paul, MN). Intraoperative epicardial ECHO confirmed antegrade LMCA flow, normal myocardial contractility, and minor fistula leak. The cross-clamp was released and normal sinus rhythm without ischemic ECG changes emerged upon myocardial reperfusion. Post-operative hemodynamic catheterization revealed high RV systolic and diastolic pressures (60/25 mmHg), LVEDP (90/26 mmHg), central venous pressure (25 mmHg), pulmonary venous hypertension (mean 30 mmHg) with increased pulmonary flow. Residual fistula leaks (Fig. 4, Additional file 4: Video 4) required interventional endovascular embolization using multiple occluding devices which were redelivered via LMCA without sequalae (Fig. 5, Additional file 5: Video 5).

At 2-year follow-up the girl was asymptomatic on pharmacotherapy (metoprolol and aspirin) with good exercise tolerance (NYHA II). A resting ECG showed normal sinus rhythm without symptoms of ischemia while 2DE revealed good EF (50%) and a sealed fistula.

## Discussion and conclusions

Ischemic ALCAO with cardiomyopathy is an indication for urgent surgery using a left internal mammary artery bypass graft, although in complex heart defects and small children heart transplant is considered a viable option. Our patient’s resting ECG was not suggestive of ischemia as suprasystemic RV pressure and wide fistula patency secured left coronary flow despite ALCAO. Yet, upon reconstructing the LMCA ostium and decompressing the RV, coronary steal phenomenon and heart failure ensued. In small children, coronary ostium reconstruction with a patch offers an alternative solution [4]. In those with a coronary fistula, traditional surgical ligation is preferential to avoid myocardial ischemia [5]. However, the fistula orifice is difficult to visualize and precisely ligate in pre-adolescent children with epicardial adipose tissue. Patched coronary ostium reconstruction and simultaneous hybrid fistula embolization are viable procedures in anomalies technically difficult to approach. Our concerns about RV decompression and coronary steal phenomenon proved to be well founded as further embolization were needed for residual fistula leakage.

Critical considerations and limitations of the presented case include shortage of published literature and guidelines for children with coronary anomalies. Rigid surgical-only solutions proposed in the literature lack flexibility to adapt to individual cases which appears to be crucial. For instance, a pragmatic approach depends on body weight, coexisting heart defects, hemodynamic flow, and the surgeon’s experience. In the presented case, the authors demonstrate the viability of a hybrid approach to complex coronary anomalies with high perioperative mortality to restore functional anatomy and physiology.

A key learning objective recalls the risk of coronary steal phenomenon appearing after hypertensive RV decompression—a vital source of coronary perfusion in patients with cameral fistulas. Complex congenital coronary anomalies with technical difficulty ought to strongly consider a novel hybrid treatment with staged planning and close hemodynamic monitoring.

## Supplementary Information

**Additional file 1: Video 1.** Aortography (antero-posterior view). The wide RCA with absent LMCA.

**Additional file 2: Video 2.** Selective fistula angiography (LAO 48° CRA 28°). Wide ventriculo-coronary fistula. LMCA ostium atresia. Collateral coronary anastomoses.

**Additional file 3: Video 3.** Selective fistula angiography. Long and wide fistula (LAO 28°, Caudal 37°). Collateral coronary anastomoses.

**Additional file 4: Video 4.** Selective LMCA angiography (antero-posterior view) with wide patent fistula and steal phenomenon into a decompressed right ventricle.

**Additional file 5: Video 5.** Selective LMCA angiography with restored coronary flow and occluded fistula.

## Data Availability

The data are available for public access.

## Abbreviations

ALCAO: Atresia of the left coronary artery ostium
EF: Ejection fraction
LMCA: Left main coronary artery
LV: Left ventricle
LVEDP: Left ventricle end-diastolic pressure
NYHA: New York Heart Association—functional classification of heart failure
RCA: Right coronary artery
PVS: Pulmonary valvar stenosis
RV: Right ventricle
RVEDP: Right ventricle end-diastolic pressure
TTE: Transthoracic echocardiogram
